# Investigating regional excess mortality during 2020 COVID-19 pandemic in selected Latin American countries

**DOI:** 10.1186/s41118-021-00139-1

**Published:** 2021-11-03

**Authors:** Everton E. C. Lima, Estevão A. Vilela, Andrés Peralta, Marília Rocha, Bernardo L. Queiroz, Marcos R. Gonzaga, Mario Piscoya-Díaz, Kevin Martinez-Folgar, Víctor M. García-Guerrero, Flávio H. M. A. Freire

**Affiliations:** 1 Universidade Estadual de Campinas, Campinas, Brazil; 2 Universidade Federal de Minas Gerais, Belo Horizonte, Brazil; 3 Public Health Institute, Pontifical Catholic University of Ecuador (PUCE) – Ecuador, Quito, Ecuador; 4 Departamento de Demografia e Ciências Atuariais, Universidade Federal do Rio Grande do Norte, Natal, Brazil; 5 Universidade Federal de Goias, Goiânia, Brazil; 6 Urban Health Collaborative & Department of Epidemiology and Biostatistics, Dornsife School of Public Health, Drexel University, Philadelphia, PA USA; 7 El Colegio de México, Mexico City, Mexico

**Keywords:** COVID-19, Pandemic, Excess mortality, Latin America, Subnational areas

## Abstract

In this paper, we measure the effect of the 2020 COVID-19 pandemic wave at the national and subnational levels in selected Latin American countries that were most affected: Brazil, Chile, Ecuador, Guatemala, Mexico, and Peru. We used publicly available monthly mortality data to measure the impacts of the pandemic using excess mortality for each country and its regions. We compare the mortality, at national and regional levels, in 2020 to the mortality levels of recent trends and provide estimates of the impact of mortality on life expectancy at birth. Our findings indicate that from April 2020 on, mortality exceeded its usual monthly levels in multiple areas of each country. In Mexico and Peru, excess mortality was spreading through many areas by the end of the second half of 2020. To a lesser extent, we observed a similar pattern in Brazil, Chile, and Ecuador. We also found that as the pandemic progressed, excess mortality became more visible in areas with poorer socioeconomic and sanitary conditions. This excess mortality has reduced life expectancy across these countries by 2–10 years. Despite the lack of reliable information on COVID-19 mortality, excess mortality is a useful indicator for measuring the effects of the coronavirus pandemic, especially in the context of Latin American countries, where there is still a lack of good information on causes of death in their vital registration systems.

**Supplementary Information:**

The online version contains supplementary material available at 10.1186/s41118-021-00139-1.

## Introduction

Several cases of COVID-19 emerged in Latin America in early 2020, following what was observed in Europe and the United States (Andrus et al., 2020; Burki, 2020; Muñoz, 2020; Rodriguez-Morales et al., 2020). In late 2020 and early 2021, Latin America was considered the epicenter of the pandemic, and several countries in the region presented the highest number of cases and deaths in the world (Gideon, 2020; Karlinsky & Kobak, 2021; Muñoz, 2020). However, proper tracking of the pandemic and investigation of its impact are limited by data quality (Croda et al., 2020; Torres & Sacoto, 2020). Countries in the region face several problems related to the pandemic: for example, until the early months of the pandemic, the amount of COVID-19 testing in the population was low (Burki, 2020; Peto, 2020). Overall, Latin America had the lowest test rates in the world, around 63 per 100,000 inhabitants; expanding tests and establishing a contact tracing scheme would be two of the most important measures to control the spread of the pandemic. There were also delays between the COVID-19 tests and the time their results were released. In some cases, it took more than 2 weeks to reveal the outputs (Bastos et al., 2020; Werneck & Carvallo, 2020). Thus, one way to measure the impact of the pandemic is to analyze excess mortality, that is, the level of mortality at a particular point in time compared to recent trends (or expected value).

In the current crisis, proper information on causes of deaths is crucial to understand the effects of the pandemic (Rios-Neto, 2021). However, one cannot expect civil registration and vital statistics (CRVS) systems to provide information on causes of death rapidly, since they must follow certain procedures that are specific to each country. In addition, in many regions of the globe, CRVS systems were disrupted during the pandemic, affecting the collection of birth and death data, especially across more vulnerable groups (AbouZahr et al., 2021). Even in more developed economies, with well-organized vital registration systems and a reduced lag in cause-of-death codification, variability due to different definitions of COVID-19 mortality make comparisons between countries difficult (AbouZahr et al., 2021; Karanikolos & McKee, 2020).

Many countries around the world developed systems to release information on all-cause mortality based on information from health departments or from the civil registration system; using information from these sources, different data sets were developed to provide data on all-cause mortality for both developed and developing countries (Karlinsky & Kobak, 2021; Németh et al., 2021). The information on all-cause mortality allows us to investigate the impacts of the COVID-19 pandemic by comparing overall levels of mortality over a recent period with mortality levels from previous years. In this context, several countries have estimated excess deaths for all causes at the national level and/or at subnational levels and compared 2020 mortality with respect to past death trends—either the past year or an average of recent years—as an effective way to evaluate the total burden of the COVID-19 pandemic. Excess mortality analysis has the advantage of including not only direct COVID-19-related mortality but also indirect deaths, and all-cause mortality are not affected by miscoding on death certificates (Beaney et al., 2020; Blangiardio et al., 2020; Vandoros, 2020).

However, most of the current publicly available data and analysis focus on the whole country and do not show subnational trends that are particularly important to understand the dynamics of the pandemic, especially in a region marked by large social and economic inequality. In this paper, we focus on the pandemic’s impact at national and subnational levels, analyzing excess mortality in selected Latin American countries and their regions and its impacts on life expectancy. The contribution of this study is that there are very few analyses looking at the subnational mortality level (Blangiardo et al., 2020; Calderón-Larragana et al., 2020; Krieger et al., 2020; Modig et al., 2021), and most of the research on excess mortality focused on national populations. Latin America is characterized by large socioeconomic and regional inequalities, and there is strong empirical evidence of high heterogeneity in mortality within and across countries (Núñez & Icaza, 2006; Palloni & Pinto-Aguirre, 2011; Peralta et al., 2019a; Piscoya-Diaz & Queiroz, 2010; Queiroz et al., 2017, 2020). Finally, in the early moments of the epidemic, a large volume of deaths was concentrated in a few places in each country, where the first infections were registered. However, over time, the pandemic moved to less-developed areas (Castro et al., 2021a; Lima et al., 2021). Several reports (Dale & Stylianou, 2020; Economist, 2021; Financial Times, 2021) show estimates of excess mortality due to COVID-19 at the country level, but few studies examine the impacts of this disease at the subnational level.

### Background: the COVID-19 pandemic in Latin America

During the COVID-19 pandemic in several countries of Latin America, government officials contradicted recommendations from the World Health Organization and avoided imposing more restrictive rules on their populations. Some countries’ leaders did not even incentivize their inhabitants to wear masks or to avoid large gatherings (Acosta, 2020; Benítez et al., 2020; Gofen & Lotta, 2021; Mundt, 2021). For example, in the early months of the pandemic, the Brazilian president, Jair Bolsonaro, encouraged people to go out, commonly advocating for relaxing social distancing measures and suggesting that COVID-19 was a regular flu. Evaluating the effects of asymmetric information on social distancing, Ajzenman et al. (2020) showed that Bolsonaro’s words and actions had a strong effect on relaxing social distancing in the municipalities of the country, which in turn might increase the spread of infection.

Similar non-application of non-pharmaceutical measures to control the pandemic by federal officials were observed in other countries of the region. The first three cases of COVID-19 in Mexico were confirmed on 27 February 2020 by the Sentinel Surveillance System^1^: two in Mexico City and one in Sinaloa. By 28 February, the Mexican Secretariat of Health announced the first phase of the pandemic in Mexico, characterized by cases imported from abroad (SS-SPPS, 2020; Suárez et al., 2020). On 18 March, the first death from COVID-19 was registered, and on 23 March, the government declared the beginning of the National Social Distancing Strategy. On 24 March, the Secretariat of Health announced the beginning of the second phase of the pandemic in Mexico, characterized by community transmission. Since 6 April 2020, the Secretariat of Health has mandated that all hospitals should administer polymerase chain reaction (PCR) tests to all severely ill patients and to 10 percent of ambulatory patients (CONAVE, 2020). Tests applied in private laboratories and private hospitals on patients who did not require hospitalization are not recorded in official registries. On 12 April 2020, the Secretariat of Health launched a publicly available dashboard listing COVID-19 cases and deaths by age, sex, and other individual variables, with daily updates.

Chile adopted a high number of daily tests, together with local and dynamic quarantines, which depend on the number of active infected cases. The country also implemented strict social isolation measures and follow-up of the infected and the people that they have been in contact with (Kiwi & González, 2020). These measures were not effective enough; Chile experienced a high number of COVID-19 cases from April 2020 on, and the country is marked by social and regional heterogeneous impacts of the pandemic (Bilal et al., 2021). In Guatemala, the first case of COVID-19 was detected on 13 March 2020. An outdated epidemiological surveillance system made it difficult to take timely actions against the pandemic (Cerón, 2019). However, several non-pharmaceutical interventions were implemented in the following days.

In the context of a pandemic, the knowledge of how COVID-19 is affecting mortality and the health status of a population became an important issue for mortality and health studies. However, reliable methods to estimate and project the pandemic’s impact on the number of deaths (and life expectancy) were limited or inaccurate. In fact, a clear death toll of the pandemic might take some time to be understood, since proper mortality registration systems do not report real-time data for most of the countries by causes of death (Appleby, 2020; Labib & Aroori, 2020). In addition, defining mortality by COVID-19 was complicated, and several places in the world underreported this cause of death (Lau et al., 2020). As an example, in Chile, for the number of recovered COVID-19 cases, there is controversy in the records due to the criteria the Ministry of Health used, which establishes as recovered from the disease any person who completes 14 days from the COVID-19 confirmation, does not present any symptoms after that period, and is found to be in good health (Guerrero-Nancuante & Manríquez, 2020). This fact may also affect the number of deaths recorded due to this illness.

However, there are appropriate methods for dealing with these issues, making it possible to get an idea of how mortality has increased at a given point in time and which ages were most affected (Felix-Cardoso et al.^2020^; Kontopantelis et al., 2021; Krieger et al., 2020). Thus, one way to estimate the dynamics of the pandemic and to measure its impact on mortality is by looking at existing death data to investigate the overall excess mortality in the year 2020 relative to the number of deaths from previous years.

It is important to emphasize that not all excess deaths are causally related to COVID-19, but the estimated excess of deaths, compared to previous years, can help to reveal trends in lethality of this disease (Adjiwanou et al., 2020; Felix-Cardoso et al. 2020; Kontopantelis et al., 2021; Krieger et al., 2020; Nogueira, 2020). As Noymer (2020), Helleringer (2020), and Karlinsky and Kobak (2021) indicate, we can consider the effect of the epidemic on four types of mortality that are reflected in excess mortality (Adjiwanou et al., 2020):

- The direct mortality effect, which is the current number of deaths recorded as COVID-19 deaths;
- The direct–indirect effects that were measured as early deaths in the occurrence of COVID-19 and that have been mistakenly recorded as influenza or other respiratory illness, so that subsequent deaths from COVID-19 are not registered as such;
- Indirect mortality, or deaths from a health problem that was not treated due to the overloading of the health system and the intensive care units dedicated to COVID-19 treatment; and
- Competing mortality risks, which represent a person who died from COVID-19 today but who would have died of other diseases in the near future, with COVID-19 being a competing risk of mortality (Karlinsky & Kobak, 2021; Santos & Howard, 2018a; Santos et al., 2018b).

During the pandemic, we will not be able to measure these four effects of this disease on mortality, but we can assess the excess number of deaths. A central question is how to measure this counterfactual: “What is the number of deaths that could occur in the absence of COVID-19?” In most cases, it is calculated by the average number of deaths over a given period in comparison with other recent years, that is, when SARS-CoV-2 did not occur as a cause of death (Adjiwanou et al., 2020; Karlinsky & Kobak, 2021).

### Data and methods

We use a variety of data sources for each country. In general, most countries in the region manage to produce and distribute the current number of overall mortality at the local level rapidly (Carrillo-Larco, 2020). However, one needs to be cautious because of the delay in reporting during the pandemic and the level of completeness. Table 1 summarizes the data sources and other important characteristics of the death information used in this study and Additional file 1 provides the Latin America map with the location from each country analyzed.

**Table 1:** Summary of death registration sources for the six selected Latin American countries in 2020

Country	Data source	Place of death registration	Area studied	Periodicity of data availability^a^	Smallest geographical coverage	Data quality^b^
Brazil	Ministry of Health	Place of residence	27 federal states	Yearly data available since 1979	Municipality level	90–99% coverage of death counts
Chile	Ministry of Health	Place of residence	16 regions	Yearly data available since 1955	Municipality level	90–99% coverage of death counts
Ecuador	National Ecuadorian Statistical Office	Place of residence	24 provinces	Yearly data available since 1961	Municipality level	80–89% coverage of death counts
Guatemala	National Registry of Persons	Place of residence	22 departments	Yearly data available since 2009	Municipality level	94% coverage of death counts
Mexico	Secretariat of Health	Place of residence	32 federal states	Yearly data available since 1955	Municipality level	90–99% coverage of death counts
Peru	Ministry of Health	Place of residence	25 departments	Yearly data available since 1966	Municipality level	50–74% coverage of death counts

^a^The year that each country started to collect death information is from the World Health Organization

^b^Based on the United Nations Demographic Yearbook Vital Statistics Questionnaire (United Nations Statistics Division) for the following years: 2017 (Brazil), 2020 (Chile), 2020 (Ecuador), 2007 (Guatemala), 2020 (Mexico), and 2015 (Peru)

All the data sources refer to deaths registered at the place of residence, and they are available yearly, but the source of death information differs by country: data may come from the ministry of health, the national statistical office, or the secretariat of health. In addition, the quality of death registration varies across the countries; with the exceptions of Peru and Ecuador, the other countries analyzed present death counts coverage above 90 percent.

For Brazil, we use data from administrative death records through death certificates (Silva et al., 2020). These data are provided by the Brazilian Ministry of Health (2021) and are publicly available on its website. Data on the most recent death dates may undergo some changes due to the entry of new records. The process for bringing the data into the system may take a few days, and corrections are made to registered events as needed (Fujiwara, 2020). That means the analysis must be updated and adjusted regularly.

For Ecuador, we use monthly civil death registers for the year 2020 that are available publicly (Gobierno de la República del Ecuador, 2021). As in the case for Brazil, this data is preliminary, because the database for civil registry identification and certification is permanently updated through death registrations made by Ecuadorian citizens. Before 2020, the level of completeness of death registration in that country did not exceed 90 percent.

Peruvian mortality data is registered by the Ministry of Health (2021). We obtained the registered deaths from January 2017 to the end of 2020. Peruvian death registration is not uniform: the level of completeness of death registration is inversely correlated with the region’s economic development (Piscoya-Diaz & Queiroz, 2010). The quality of deaths recording has increased in the last 10 years, but there is still room to improve its completeness. According to estimates from the United Nations (2021) in Table 1, death registration coverage was below 80 percent in years preceding the COVID-19 pandemic. The microdata contains information about deaths by place of residence, date of death, age, sex, and principal cause of death.

In Chile, death count data were compiled through the Chilean Ministry of Health (2021) (Departamento de Estadísticas de Información de Salud). This data relates to the weekly death information provided by the Chilean Ministry of Health and National Statistical Office for 346 municipalities; it is constantly updated by Chilean statistical offices.

Guatemalan daily mortality counts were obtained from the National Registry of Persons. This data is available by request from its website (Registro Nacional de las Personas - RENAP, 2021). The data are collected in each of the 22 departments. By law, all deaths should be registered prior to a funeral, but there are delays between the occurrence of the death and the registering of it (this varies between departments and municipalities).

Mexican death counts were taken from the open access dashboard to analyze the excess mortality of Mexico due to COVID-19, with data provided by the Secretariat of Health (DGE-SS, 2021). For 2015 to 2019, the information is available from the vital statistics provided by the National Institute of Statistics and Geography (INEGI, 2020).

For the geographical analysis, the subnational areas considered here are like states, with different definitions in each country. In Brazil, we selected 27 Brazilian federation units (26 states and the federal district, Brasília). In Mexico, we selected data from 32 states. In the other countries, we selected data from the following geographical areas: 16 Chilean regions, 24 Ecuadorian provinces, 22 Guatemalan departments, and 25 Peruvian departments.

The choice for these geographical aggregations is justified to avoid the problem of population mobility and deaths that could be registered in neighboring areas. To reduce this issue and the confusion between deaths registered in the place of residence versus place of occurrence, the strategy adopted was to work with administrative areas that still show a picture of excess mortality diversity within studied countries, but with regions that are large enough in terms of population size and area. For example, in the case of Brazil, we chose federal states, which show small migration flows between these areas over the past few years; population mobility has become a short-distance phenomenon, from urban to urban places (Lima & Braga, 2013). The same could be said about Chile, Ecuador, Guatemala, Mexico, and Peru (Guzmán et al., 2006; Pérez-Campuzano et al., 2018; Pontarollo & Segovia, 2019; Yamada, 2010).

### *P*-score estimate of excess mortality

We estimate excess mortality by estimating *P*-scores, as defined by Ritchie et al. (2020) and explained in detail in Muellbauer and Aron (2020). We used average monthly death data from 2015 to 2019 and compared it with the number of deaths from January to December 2020 for each country and its subnational areas. The only exception is Peru, where we do not have death registers before 2017; in this case, we estimate average deaths from 2017 until 2019 (Muellbauer & Aron, 2020). The *P*-score is defined as.

1 $$P - {\text{score}}\, = \,\left[ {\left( {{\text{Deaths}}_{{{\text{Period}}\# {2}0{2}0}} {-}{\text{Average Deaths}}_{{{\text{Period}}\# {2}0{15}{-}{2}0{19}}} } \right)/{\text{Average Deaths}}_{{{\text{Period}}\# {2}0{15}{-}{2}0{19}}} } \right]\, \times \,{1}00$$

*P*-score is a useful measure, but it has limitations. The 5-year average death could be seen as a relatively crude measure of expected mortality, because it does not account for trends in population size or mortality (Ritchie et al., 2020; Muellbauer & Aron, 2020). For instance, in areas that have an increasing trend in mortality, a 5-year average will overestimate excess deaths, while for localities that have a decreasing trend in mortality, it will underestimate excess deaths. Despite this limitation, when we look at the development of deaths in the past 5 years, in all countries and regions considered, mortality did not show an expressive trend (decrease or increase in mortality) over time.

It is also possible to investigate excess mortality by age using *P*-scores, as looking at age-specific mortality is important, specifically when our concern is to understand how COVID-19 affects mortality trends. However, mortality data sets organized by age are not always available in Latin American countries. In addition, the main concern of this paper is to study excess mortality in subnational areas of Latin American countries, something that has not been addressed by the current studies of excess mortality that focus only on countries.

There is some uncertainty with excess mortality, because not all countries have the infrastructure and capacity to register and report all deaths, and delays in death reporting make mortality data provisional and incomplete in the weeks, months, and even years after a death occurs. To cope with the uncertainty of *P*-score estimates, we applied Markov chain Monte Carlo, with random sampling and replacement for the deaths counted in 5 years before 2020, simulating this procedure with sampling and replacement 1000 times. We estimate the distribution of *P*-scores to years before 2020 and compare the last *P*-scores before the pandemic with the ones including 2020 (during the pandemic). The only assumption is that the monthly death counts in each region, between the years before the COVID-19 pandemic, are randomly distributed.

The problem of the underreporting of deaths is less problematic; we assume that this issue did not change much during the past 5–6 years. However, later death registrations could still affect the estimates of excess mortality downwards: this happens if we assume that the registration systems could be negatively affected by the COVID-19 crisis. To minimize the later registration of events, we used the most up to date death information for each country (Additional files 1, 2).

### Life expectancy estimates considering the effects of excess mortality

To measure the effects of excess mortality on life expectancy, we used a simple methodological approach that differs from Aburto et al. (2021a) and Castro et al. (2021b). The basic idea is to use life table entropy H (Demetrius, 1974; Fernandez & Beltrán-Sánchez, 2015; Keyfitz, 1977) and the proportion of excess mortality in 2020 in each country and see how these two measures affect current life expectancy. The entropy of the life table describes the association between the relative changes in life expectancy with changes in age-specific mortality rates (Demetrius, 1974), and the *P*-scores measure the proportion that mortality in time *t* and region *y* exceeds its usual level in comparison with the mortality in *y* in time t*-1* (Giattino et al., 2021). These two measures in combination will give the amount of countries’ loss in life expectancy at birth in 2020.

Given the survival function *l(x)* of each country, in discrete terms, entropy was estimated as

2 $$H = \left( {\frac{{\mathop \sum \nolimits_{x = 0}^{w + } l\left( x \right)ln(l\left( x \right))}}{{\mathop \sum \nolimits_{x = 0}^{w + } l\left( x \right)}}} \right)$$

Hence, considering all measures, and assuming that we know for each country the presumable life table for the year 2020 (we defined it as 2020"), the loss in life expectancy at birth for that year is estimated as

3 $$\varphi^{{e_{0}^{2020} }} = P_{score} \times H^{2020} \times e_{0}^{2020}$$

where the term $\varphi^{{e_{0}^{2020} }}$ indicates the loss in life expectancy at birth in 2020, *Pscore* measures the excess of mortality in 2020, and $H^{2020}$ and $e_{0}^{2020}$ indicate, respectively, the presumable life table entropy and life expectancy at birth in 2020. Unfortunately, we did not have official life tables estimates for all countries and their regions during the pandemic. Thus, to keep the analysis uniform, we use a country’s life tables estimated by the United Nations (2019) in its Revision of world population prospects. In that document, the last available life table before 2020 is for 2018. We assumed that survival functions did not change much in those 2 years, and so the mortality levels of 2018 are a good approximation for the life table that we would have if the pandemic had not happened in 2020. Another shortcoming here is that we did not have recent life tables for all the regions in each country, so we present estimates for the national level.

## Results

**Fig. 1:**
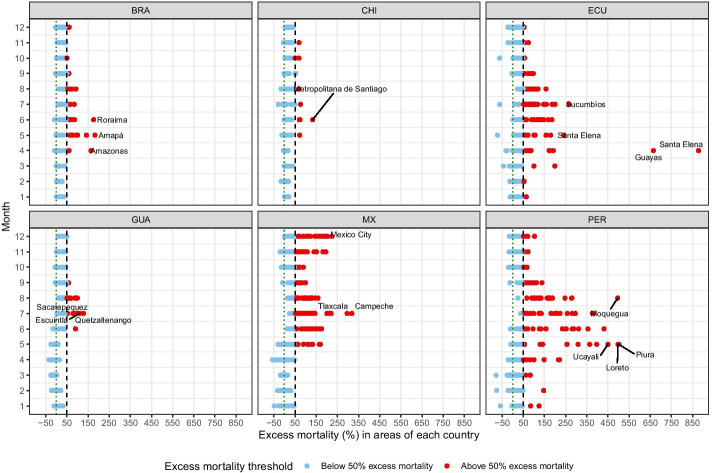
Excess mortality during the COVID-19 pandemic: deaths from all causes and ages compared to previous years in selected Latin American countries and their subnational areas in 2020. Threshold of 50 percent excess mortality. Source: Ministry of Health of Brazil, Chile, and Peru, 2021; National Civil Registry Ecuador (Gobierno de la República del Ecuador, 2021); National Registry of Persons Guatemala (RENAP, 2021); and Secretariat of Health, Mexico (DGE-SS 2021). Names and acronyms of countries are presented in Additional file 4: Table S1

Figure 1 shows, for the selected countries and their subnational areas, the percentage of excess mortality according to the estimates of the *P*-scores (Additional file 2 also provides other visualization of excess mortality in the selected countries). The interpretation of this measure is straightforward: where a country shows a *P*-score of 100 percent in a given month of 2020, for example, that means the death count for that month was 100 percent higher—that is, double—than the average death count in the same month over the previous 5 years. In Additional file 3: Figure S3. We present the uncertainty estimates for excess mortality in each subnational area. Considering the whole year, in very few areas (eight in total), excess mortality was not certain to have occurred.^2^

To facilitate visualization, we differentiate the areas by a cut-point of 50 percent excess mortality. We show the results for each country and its regions during all months of 2020. The findings indicate notably high increases in mortality in many regions of each country. The timing and magnitude of excess mortality also vary across countries and regions. For example, Brazil presented excess mortality starting in April 2020, with peaks of excess mortality in many of its regions from May to August, but with these numbers reduced in the following months of the second half of 2020. Chile showed a development similar to Brazil: some regions presented excess mortality in May and June, and many other locations in this country showed *P*-scores above 50 percent in the second half of 2020.

In Ecuador, excess mortality was notably high in the first months of 2020. Two regions of this country caught our attention, where the *P*-score reached values above 600 percent in April. That is, death counts in that month were more than six times the average death counts in the same month over the previous 5 years. Many other locations in this country also presented excess mortality in several months of the past year.

In Guatemala, excess mortality was more visible from June to August. From September on, *P*-scores dropped considerably. Mexico and Peru show a similar development; many regions presented excess mortality peaking at different moments of 2020. In Mexico, for example, the first peak of excess mortality was May, then July, then at the end of the year, giving evidence of a possible second wave of the pandemic there. Peru had more variability in terms of excess mortality throughout 2020. We saw huge peaks of excess mortality between from May until August and, like in Mexico, in the last months of 2020, the *P*-score started to increase again.

**Fig. 2:**
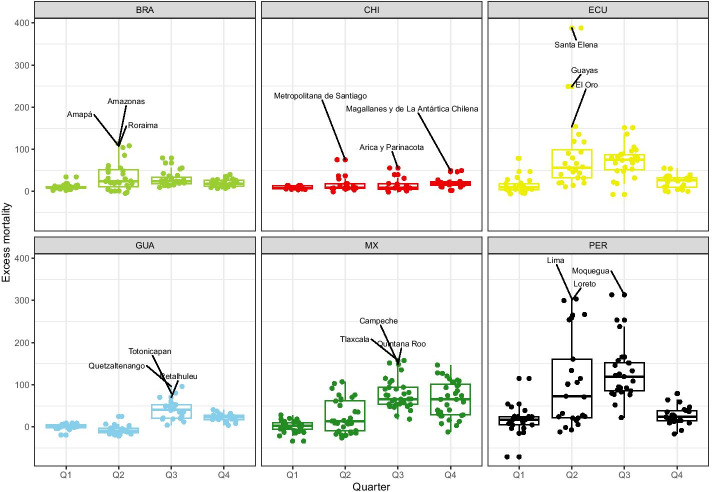
Distribution of excess mortality during the COVID-19 pandemic: deaths from all causes compared to previous years, all ages in selected countries and regions of Latin America in 2020. Source: Ministry of Health, Brazil, Chile, and Peru, 2021; National Civil Registry Ecuador (Gobierno de la República del Ecuador, 2021); National Registry of Persons Guatemala (RENAP, 2021); and Secretariat of Health, Mexico (DGE-SS, 2021). Names and acronyms of countries are presented in Additional file 4: Table S1

Figure 2 shows the dispersion of excess mortality in each country, considering each quarter of 2020. Many subnational areas—concentrated in Ecuador, Mexico, and Peru—have *P*-scores above 50, indicating that excess mortality in 2020 was 50 percent higher than the average value of the previous 5 years in those subnational areas. These same three countries also presented more dispersion in terms of *P*-scores and many outlier regions (especially in Peru and Ecuador) with high values of excess mortality. In Peru and Ecuador, the dispersion in excess mortality is more pronounced especially between the second and third trimesters.

In Brazil, Chile, and Guatemala, despite the excess mortality being less pronounced, the median *P*-score for these countries was still high. In Brazil, for example, considering the whole of 2020, half of the Brazilian states presented an excess mortality over 25 percent, and some outlier states had mortality close to 50 percent or higher in 2020 compared with the previous 5 years. In Brazil, more dispersion in excess mortality is observed in the second trimester, while in Guatemala, the third trimester is when the *P*-scores reached high values and some outlier areas appeared. In addition, 26 of the 32 Mexican states spent more than 3 months at over 50 percent excess mortality, and seven states spent at least 1 month at over 100 percent excess mortality. Mexico also shows great dispersions in excess mortality at different moments of the year and, from the second trimester on, the *P*-score values are considerably higher in many areas of the country. Chile is the only country analyzed that does not show much dispersion variation in excess mortality during the year.

**Fig. 3:**
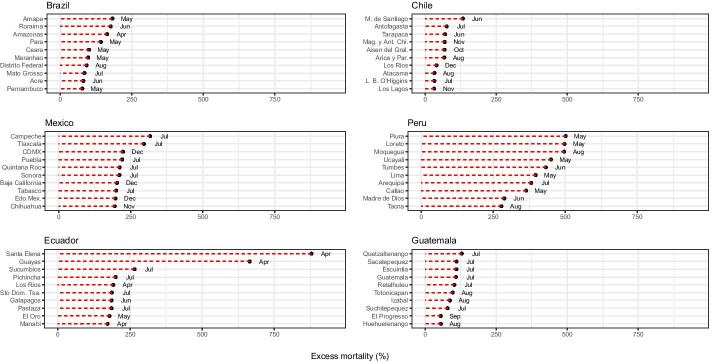
Excess mortality during the COVID-19 pandemic: deaths from all causes compared to previous years, all ages. The 10 highest values of excess mortality in regions of Latin American countries in 2020. Source: Ministry of Health, Brazil, Chile, and Peru, 2021; National Civil Registry Ecuador (Gobierno de la República del Ecuador, 2021); National Registry of Persons Guatemala (RENAP, 2021); and Secretariat of Health, Mexico (DGE-SS, 2021). Names and acronyms of countries are presented in Additional file 4: Table S1

Figure 3 provides a more detailed analysis of excess mortality by looking at selected subnational areas for each country. We selected 10 areas, according to the highest *P*-score of each country, and identified the month when mortality exceeded the expected common values. In Brazil, for instance, the highest peaks of mortality usually occurred in the month of May in five states. Eight out of ten of the federal states with the highest excess mortality were in the northern and northeastern regions of the country, where the city Manaus (North Amazon) is located. These subnational areas are historically less developed than other regions of the country (Castro et al., 2021a; Lima et al., 2021). According to Castro et al. (2021b), overall life expectancy in Brazil will be reduced by 1.3 years in 2020, and some states in the northern part of the country observed declines greater than 3 years, reaching levels observed before 2010.

In Chile, excess mortality varied a lot across the regions in 2020, with Metropolitan Santiago and Antofagasta showing the highest values of *P*-score in June and July, followed by Tarapacá. However, other regions also exceeded mortality levels at the end of last year. For example, we can mention Magallanes y la Antártica Chilena (in November 2020), Aysén del General Carlos Ibáñez del Campo (in October 2020), and Los Lagos and Los Ríos (in November and December 2020), respectively.

In Mexico, July was the highest point of the pandemic’s first wave: six states had more than 200 percent excess mortality, while Campeche was the only state with more than 300 percent excess mortality. Figure 3 also shows that the most affected Mexican states in the first wave were Campeche, Tlaxcala, Quintana Roo (where Cancun is located), Tabasco, and Sonora. These states lost more than 2 years of life expectancy in 2020 without considering excess mortality (Garcia-Guerrero et al., 2021). The most affected states at the beginning of the second wave were Mexico City (CDMX), Baja California (a border state with the U.S., where Tijuana is located), Estado de Mexico (Edo.Mex.), and Chihuahua.^3^ According to Garcia-Guerrero et al. (2021), by the end of 2020, CDMX and Baja California had lost more than 4 years of life expectancy without considering excess mortality. Taking into account excess mortality, CDMX lost more than 7 years of life expectancy for both sexes by the end of 2020 (Garcia-Guerrero et al., 2021).

In Ecuador, the pandemic started in the coastal areas and rapidly spread to the interior of the country (Torres & Sacoto, 2020). Levels of excess mortality are noticeably clear and more intense in the region of Guayas, where the port of Guayaquil is located, but the high levels of excess mortality are also observed in other coastal western parts of the country, such as Santa Elena, where excess mortality reached almost 900 percent in April 2020. Mortality explosions occurred in March and April first in coastal provinces, but epidemic and mortality hotspots gradually moved to the rest of the country. For example, in the province of Pichincha, where the capital city of Quito is located, the number of deaths has been steadily increasing since March. As Peralta et al. (2019b) mention, the most socioeconomically deprived cantons of the country are in the Amazon, Central Andean, and Northern and Central coastal regions.

In the case of Peru, we observed a considerable increase in the number of deaths compared with the years before 2020. This is the only country, where all 10 selected regions presented excess mortality above 250 percent. Three of these regions—Piura, Loreto, and Moquegua—reached 500 percent excess mortality as compared to the previous 5 years. The timing of these peaks of mortality varies throughout 2020. For example, Piura and Loreto showed these high *P*-score values in May 2020, while Moquegua presented excess mortality levels 3 months later. This empirical exercise also indicates huge excess mortality in the country’s capital, Lima (Schwalb & Seas, 2021).

For Guatemala, the results indicate less excess mortality compared to other countries. The mortality peak was reached during July in major cities, such as Guatemala City, Quetzaltenango, and Escuintla. Excess mortality was observed in subsequent months in other departments, suggesting that more excess mortality was found in cities (Pearson et al., 2021). The authors argue that levels of mortality due to the pandemic in Guatemala were underestimated because of low testing numbers and low data quality. They also suggested that most rural areas were less exposed to the pandemic. However, most of the labor market in the country is informal, and a large percentage of the population lives in vulnerable conditions, which might impact the effects of the pandemic (Rosada & Bruni, 2009; Soto, 2018).

Table 2 shows estimates of the impact of the pandemic on life expectancy at birth after considering the burden of excess mortality observed in 2020. We compare our estimates of loss in life expectancy at birth with other studies as a way to control if these diverge more than one should expect. These estimates are useful for pointing out the negative impact of the pandemic. Life expectancy at birth estimates are a simple summary of current mortality, assuming that age-specific rates remain constant for a long period. Excess mortality in 2020 is a temporary shock and will not hold into the future (Aburto et al., 2021b; Heuveline, 2021). Under normal conditions, changes in mortality tend to be steady, making its interpretation more direct; under a temporary shock (the pandemic), its interpretation might be misleading (Aburto et al., 2021b; Heuveline, 2021). In any event, based on available data, we use the impacts on life expectancy at birth, considering its limitations, to show the impact of excess mortality in each country of the region in 2020.

**Table 2:** Estimated loss in life expectancy in 2020 due to excess mortality in selected Latin American countries

Country	Assumed overall life expectancy at birth in 2020 without the occurrence of the pandemic^a^	Loss in life expectancy at birth in 2020 due to excess mortality	Corresponding year to new life expectancy at birth after discounting excess mortality of 2020
Brazil	75.56	2.42	2009
Chile	79.96	1.94	2007
Ecuador	76.70	7.91	1990
Guatemala	73.94	2.26	2011
Mexico	74.98	5.54	1987
Peru	76.37	10.91	1989

Sources: Ministry of Health, Brazil, Chile, and Peru, 2021; National Civil Registry Ecuador (Gobierno de la República del Ecuador, 2021); National Registry of Persons Guatemala (RENAP, 2021); and Secretariat of Health, Mexico (DGE-SS, 2021). United Nations, World Population Prospects 2019, https://population.un.org/wpp

^a^Considering in 2020 the same mortality levels as estimated for the year 2018 by World Population Prospects

Using Brazil as a baseline for comparison, our estimates give 2.4 years of loss in overall life expectancy at birth. Using a different approach, Castro et al. (2021b) estimated 1.3 years of reductions in life expectancy in 2019 to 2020. One must bear in mind that we used different life tables and methods, and thus small differences between estimates should be expected.

The negative impact on life expectancy at birth varies a lot across the countries in the region. Brazil, Chile, and Guatemala present the smallest reductions in overall life expectancy when compared with the other three countries analyzed. In these last countries, the expected reductions fluctuated around 2 years lost. When we consider Mexico, Ecuador, and Peru, the losses in life expectancy at birth are even higher, with values of 5.5, 7.9, and 10.9 years, respectively. These reductions are reflected in the high excess mortality numbers that these countries show across their regions. In Peru, for example, excess mortality for the whole country in 2020 was around 95 percent more deaths than the average mortality registered in previous years. The COVID-19 pandemic has negatively impacted the longevity of these three countries, with repercussions similar to those of the 1918 influenza pandemic on the United States, when we observed a reduction in life expectancy by 7–12 years (Smith & Bradshaw, 2006).

These setbacks in life expectancy are remarkable when we look at the corresponding year to the new life expectancy at birth after discounting the excess mortality estimated in 2020. In some countries, the impact of the COVID-19 pandemic has brought the country to levels of mortality of 9 years earlier (such as in Guatemala), but in other cases, the mortality has returned to levels of 30–33 years earlier (in Ecuador, Mexico, and Peru).

## Conclusions and discussion

In this paper, despite data limitations, we show that available data on all-cause mortality gave public health officials an opportunity to analyze the dimensions of the pandemic in most Latin American countries, and we do not observe a homogeneous pattern. This paper contributes to the discussion by investigating the impacts of the pandemic at the subnational level, considering regional and socioeconomic differences in the region, and focusing on excess mortality, its regional variations, and impacts on life expectancy.

We found that the most-developed regions of the countries observed the initial increase in excess mortality. This result is expected, since tourism and business travel, especially from Europe and the United States, are concentrated in those areas. Over time, we found that even with a younger population, many locations in the region observed much higher overall excess mortality compared to other countries, which might be related to poorer health and socioeconomic conditions. For example, since 2006, Mexico has been hit by a violence epidemic, due to the Mexican government’s war on drugs. That war has increased the number of homicides, and life expectancy has stagnated (Aburto et al., 2016; Canudas-Romo et al., 2014, 2016). This explains excess mortality in the first 3 months of 2020 (as described in our analyses). In Peru, the empirical exercise indicates excess mortality in the most populated area of the country—the capital, Lima—but also in many other regions. Guatemala was the least affected by the pandemic: it implemented several policy measures in early March 2020, such as school closures, mandatory use of face masks, mobility restrictions, and prohibition of public events. Guatemala also invested to increase the number of beds per 100 people as well as the number of medical staff early in the pandemic (Allin et al., 2020). Until June 2020, a decrease in overall mortality was found, following an increasing trend reaching excess mortality during consecutive months.

In the early periods of social isolation, COVID-19 containment measures may also affect overall mortality rates, due to impacts, such as reduced road accidents during lockdown, reduced violence (homicides), increased femicides (if domestic violence spiked during quarantine), or increased deaths due to other health-related issues (if hospitals became overwhelmed and health-seeking behavior changed) (López-Calva, 2020). The current database does not allow us to observe this last impact, since the civil registry does not disclose cause of death with this level of detail so quickly. On the other hand, the rate at which people die from other causes may also increase, due to greater difficulty in accessing health services, less health care, increased stress due to isolation, and other factors.

The final impacts of the pandemic on life expectancy and overall health conditions are yet to be understood. In this paper, we provided estimates of the impacts of excess mortality on life expectancy using formal demographic methods. Our estimates show an adverse impact ranging from 1.94 years in Chile to 10.91 years in Peru. However, these estimates should be considered with caution; due to data limitations, it is not straightforward to have a clear picture of the number of deaths by COVID-19 or of the accuracy of the data.

The evidence for the impacts of mortality raises important questions that can be addressed with analysis beyond that of excess mortality. In this paper, we focus on all-causes and all-ages excess mortality, but age groups, race, sex, and other socioeconomic variables should also be investigated (Wrigley-Field, 2020). It is important as well to address the impact of the pandemic by looking at its effects on the evolution of life expectancy (Aburto et al., 2021a), the variability of age at death, decomposition changes in mortality by age group, and the evolution of life expectancy (Aburto et al., 2021b).

This analysis must be constantly updated, and the data evaluated and analyzed carefully. The main caveat is the quality of information, but despite data limitations, all the estimates indicate extremely high levels of excess mortality. It is important that agencies release data in as much detail as possible—sex, age, causes of death—to allow health managers to monitor the potential effect of the pandemic on the general health of the population.

Despite improvements in CRVS systems across the region in the past few years (Glei et al., 2021; Peralta et al., 2019a; Queiroz et al., 2020), the quality of registration varies by gender and across regions. The COVID-19 pandemic may have exacerbated the differences in and impact of the overall quality of the system. AbouZahr et al. (2021) point out that in several countries, CRVS systems were not considered essential activities and were interrupted or disrupted for a certain period; they were also affected by budget constraints and movement restrictions.

The pandemic highlighted the importance of up-to-date health information to provide public health and government officials with the proper mechanisms to track the disease and evaluate its impact more broadly. Not only is the overall quality of the CRVS system important, measured in terms of coverage and completeness, but also up-to-date and timely information is needed. Niamba (2021) pointed out that in most developing countries, the system depends on person-to-person relations; during periods of crisis (social, health, or economic), these relationships might be affected.

In the future, to keep CRVS systems operating in periods of turbulence as a key tool to track the health and demographic impacts of the crisis, government officials should guarantee such services as essential and should support and protect their workers. Alternative ways of reporting vital events, using the internet, should be developed. In addition, other data sources should be considered, such as surveys conducted by mobile phones and by the national statistical office to increase the flow of information for public health planning (Fu & Schweinfest, 2020).

## Supplementary Information

**Additional file 1: Fig. S1.** Map of the Americas and the Latin American countries analyzed. Source: Own Elaboration.

**Additional file 2: Fig. S2.** Excess mortality due to the COVID-19 pandemic: Deaths from all causes compared to previous years, all ages in selected countries of Latin American countries during 2020. Source: Ministry of Health, Brazil, Chile, and Peru, 2021; National Civil Registry Ecuador (Gobierno de la República del Ecuador, 2021); National Registry of Persons Guatemala (RENAP, 2021); and Secretariat of Health, Mexico (DGE-SS,^2021^).

**Additional file 3: Fig. S3.** Confidence intervals for overall excess mortality in the Latin American countries and their regions in 2020. Source: Ministry of Health, Brazil, Chile, and Peru, 2021; National Civil Registry Ecuador (Gobierno de la República del Ecuador, 2021); National Registry of Persons Guatemala (RENAP, 2021); and Secretariat of Health, Mexico (DGE-SS, 2021). Note: Due to scale problems, we have removed the Peruvian region of Lambayeque from the graph charts. The 2020 excess of mortality in this region was 47.57, with 95 percent CI -89.65 and 865.91, indicating that the excess mortality was uncertain to have occurred.

**Additional file 4: Table S1.** List of acronyms of each country studied.

## Data Availability

The data sets analyzed during the current study are publicly available at: https://opendatasus.saude.gov.br/; https://deis.minsal.cl/#datosabiertos; https://www.registrocivil.gob.ec/cifras/; https://www.minsa.gob.pe/defunciones/; http://www.dgis.salud.gob.mx/contenidos/basesdedatos/da_exceso_mortalidad_mexico_gobmx.html; https://www.renap.gob.gt/solicitud-de-informacion-publica-decreto-57-2008v.

## Footnotes

1 The Sentinel Surveillance System was launched in 2009 for the H1N1 pandemic.

2 These areas are the Mexican state of Nayarit; the Peruvian departments of Ayacucho, Cusco, and Lambayeque; the Guatemalan departments of Alta Verapaz, Chiquimula, and Jalapa; and the Ecuadorian province of Galápagos.

3 Mexico City and the Estado de Mexico are two different and neighboring states of Mexico. Mexico City is the capital of the country.
