# Supplementary figures and images for: Investigating regional excess mortality during 2020 COVID-19 pandemic in selected Latin American countries
Source: Genus. 2021 Nov 3;77(1):30. doi: 10.1186/s41118-021-00139-1 (PMC8564791; doi:10.1186/s41118-021-00139-1)

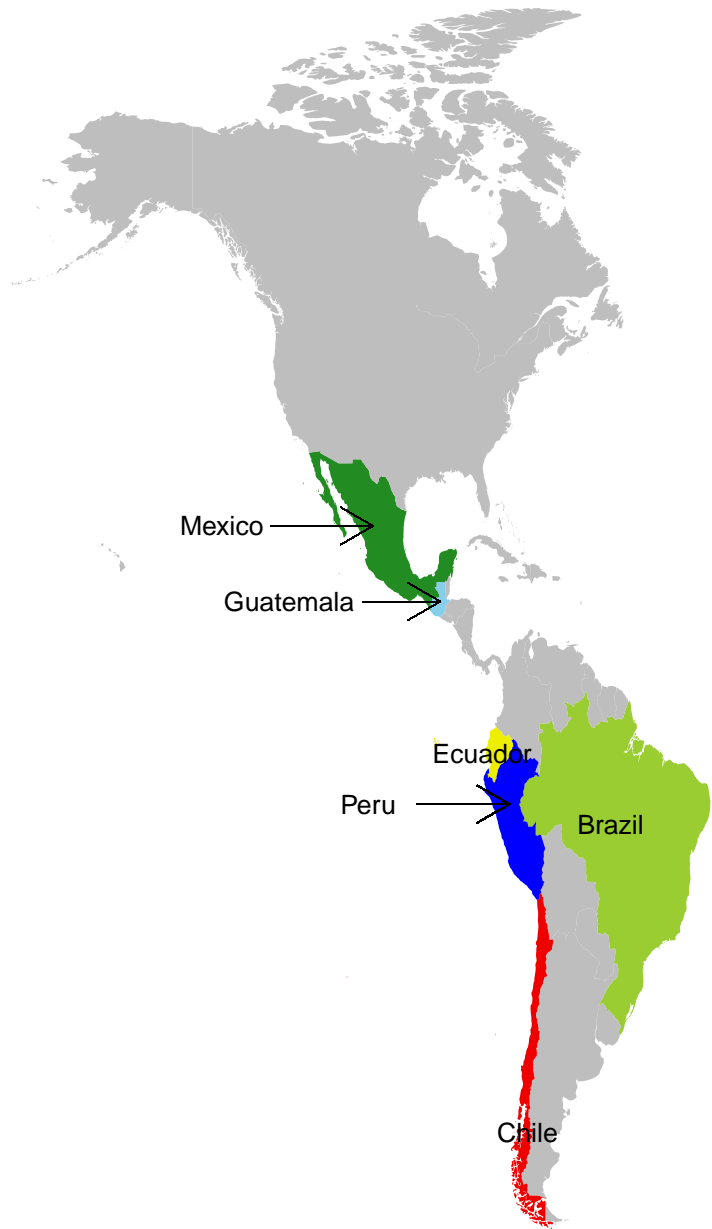

Mexico

Guatemala

Ecuador

Peru

Brazil

Chile

Supplement: Supplementary file 1 — Additional file 1: Fig. S1. Map of the Americas and the Latin American countries analyzed. Source: Own Elaboration. [file 41118_2021_139_MOESM1_ESM.pdf]

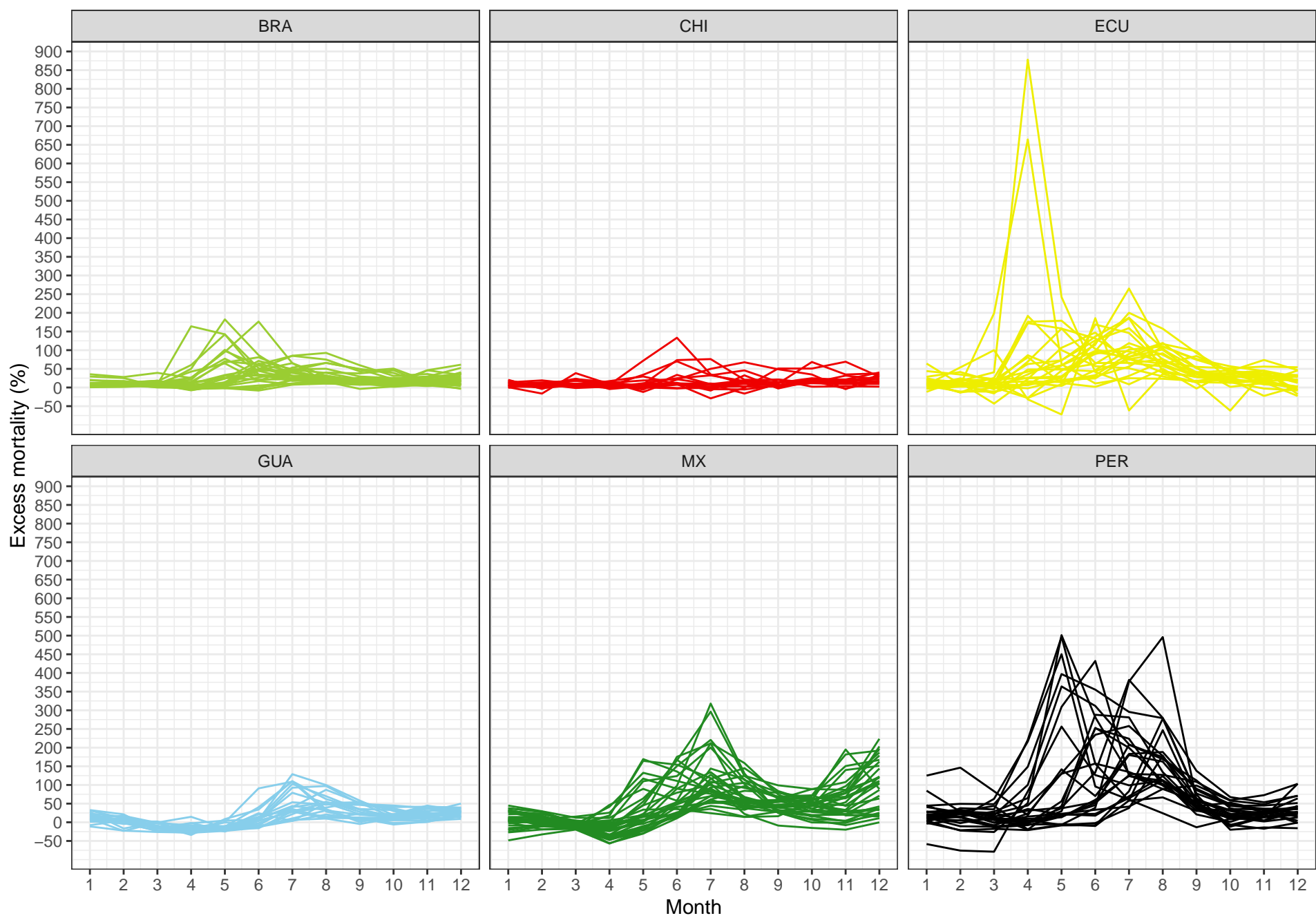

Supplement: Supplementary file 2 — Additional file 2: Fig. S2. Excess mortality due to the COVID-19 pandemic: Deaths from all causes compared to previous years, all ages in selected countries of Latin American countries during 2020. Source: Ministry of Health, Brazil, Chile, and Peru, 2021; National Civil Registry Ecuador (Gobierno de la República del Ecuador, 2021); National Registry of Persons Guatemala (RENAP, 2021); and Secretariat of Health, Mexico (DGE-SS,2021). [file 41118_2021_139_MOESM2_ESM.pdf]

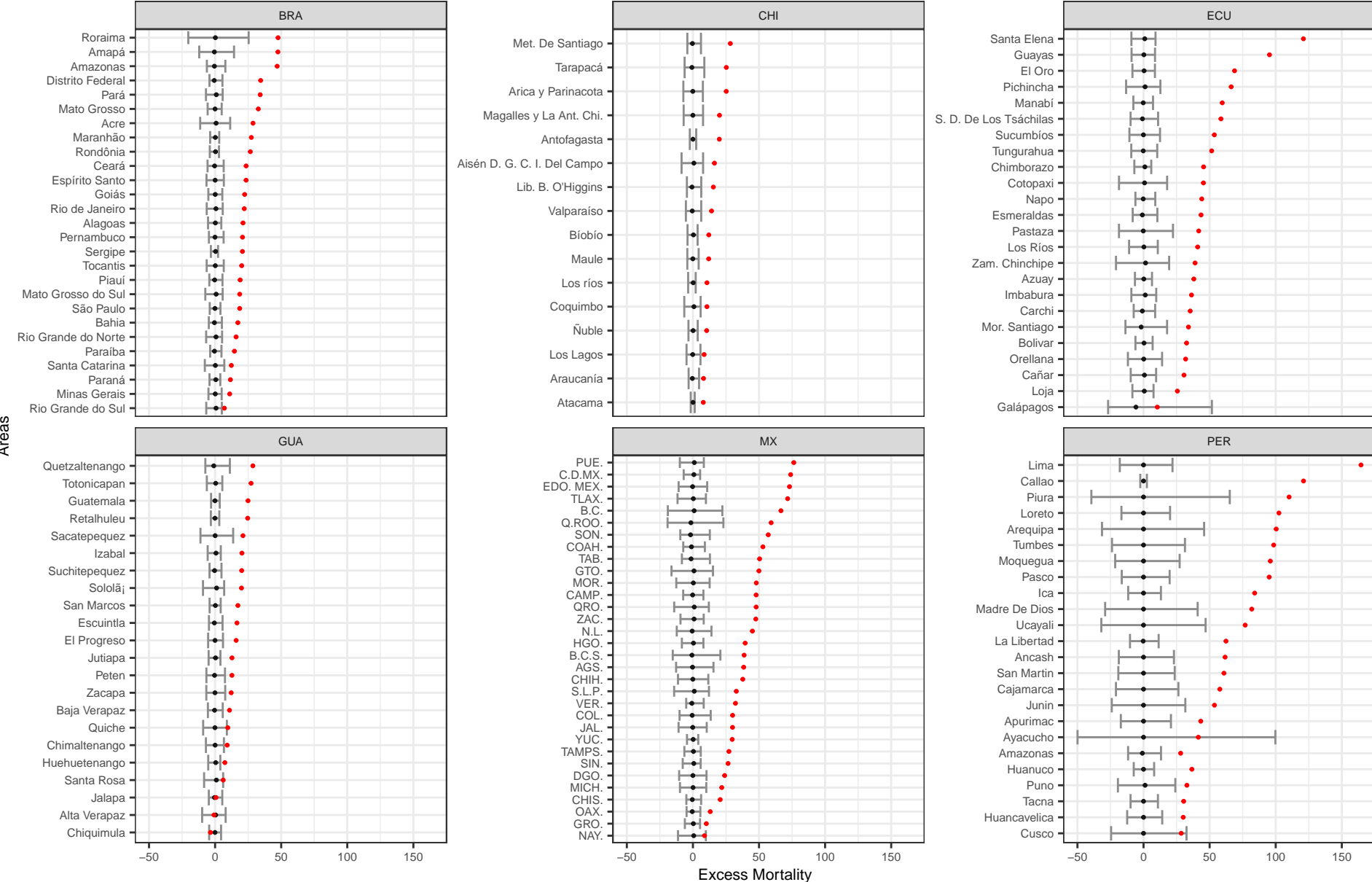

Supplement: Supplementary file 3 — Additional file 3: Fig. S3. Confidence intervals for overall excess mortality in the Latin American countries and their regions in 2020. Source: Ministry of Health, Brazil, Chile, and Peru, 2021; National Civil Registry Ecuador (Gobierno de la República del Ecuador, 2021); National Registry of Persons Guatemala (RENAP, 2021); and Secretariat of Health, Mexico (DGE-SS, 2021). Note: Due to scale problems, we have removed the Peruvian region of Lambayeque from the graph charts. The 2020 excess of mortality in this region was 47.57, with 95 percent CI -89.65 and 865.91, indicating that the excess mortality was uncertain to have occurred. [file 41118_2021_139_MOESM3_ESM.pdf]
