# Supplementary material for: Investigating regional excess mortality during 2020 COVID-19 pandemic in selected Latin American countries
Source: Genus. 2021 Nov 3;77(1):30. doi: 10.1186/s41118-021-00139-1 (PMC8564791; doi:10.1186/s41118-021-00139-1)
Supplement: Supplementary file 4 — Additional file 4: Table S1. List of acronyms of each country studied. [file 41118_2021_139_MOESM4_ESM.docx]

**Table A1. List of acronyms of each country studied.**

| **Country** | **Acronym** |
| --- | --- |
| Brazil | BRA |
| Chile | CHI |
| Ecuador | ECU |
| Guatemala | GUA |
| Mexico | MX |
| Peru | PER |

Source: Own Elaboration.
